# Pathogenicity of the H1N1 influenza virus enhanced by functional synergy between the NP^V100I^ and NA^D248N^ pair

**DOI:** 10.1371/journal.pone.0217691

**Published:** 2019-05-31

**Authors:** Woo-Jong Kim, Kye-Yeon Hur, Han Wook Park, Seung-Woo Lee, Joo-Yeon Yoo

**Affiliations:** 1 Department of Life Sciences, Pohang University of Science and Technology (POSTECH), Pohang, Republic of Korea; 2 Department of Integrative Biosciences & Biotechnology, Pohang University of Science and Technology (POSTECH), Pohang, Republic of Korea; 3 Organelle Network Research Center, Pohang University of Science and Technology (POSTECH), Pohang, Republic of Korea; University of Iowa, UNITED STATES

## Abstract

By comparing and measuring covariations of viral protein sequences from isolates of the 2009 pH1N1 influenza A virus (IAV), specific substitutions that co-occur in the NP-NA pair were identified. To investigate the effect of these co-occurring substitution pairs, the V100I substitution in NP and the D248N substitution in NA were introduced into laboratory-adapted WSN IAVs. The recombinant WSN with the covarying NP^V100I^-NA^D248N^ pair exhibited enhanced pathogenicity, as characterized by increased viral production, increased death and inflammation of host cells, and high mortality in infected mice. Although direct interactions between the NP^V100I^ and NA^D248N^ proteins were not detected, the RNA-binding ability of NP^V100I^ was increased, which was further strengthened by NA^D248N^, in expression-plasmid-transfected cells. Additionally, the NA^D248N^ protein was frequently recruited within lipid rafts, indirectly affecting the RNA-binding ability of NP as well as viral release. Altogether, our data indicate that the covarying NP^V100I^-NA^D248N^ pair obtained from 2009 pH1N1 IAV sequence information function together to synergistically augment viral assembly and release, which may explain the observed enhanced viral pathogenicity.

## Introduction

The genome of influenza A virus (IAV), a member of the *Orthomyxoviridae* family, consists of a segmented negative-sense single-strand RNA, and changes in the influenza viral genome are frequent events due to the accumulation of mutations and reassortment of RNA segments [1]. In addition to seasonal flu, IAV pandemic infections occasionally occur, with severe impacts on public health and society. There have been extensive efforts using various approaches to understand the nature of influenza pandemics. Among them, sequence comparison and reverse genetics using recombinant virus in murine or primate systems have been useful for identifying key RNA segments or sequence substitutions that contribute to influenza viral pathogenicity.

The 2009 pandemic H1N1 influenza A virus (2009 pH1N1 IAV) emerged and spread rapidly [2]. Although the 2009 H1N1 pandemic flu appeared to be mild compared to the 1918 H1N1 Spanish flu or the 1968 H3N2 Hong Kong flu, it exhibited atypical pathological potential distinct from that of seasonal influenza, with an exceptionally rapid rate of spread [3]. Sequence comparison between 2009 pH1N1 IAV and other pandemic strains has been performed to characterize amino acid substitutions contributing to pathogenicity. For example, the HA2 E47K substitution in hemagglutinin (HA) of the 2009 pH1N1 IAV reduces the pH threshold for membrane fusion, conferring the virus with thermal stability and infectivity, which partially explains its rapid spread and adaptation to humans [4]. Similarly, 2009 pH1N1 IAV substitutions in neuraminidase (NA) allow for low-pH stability [5]. Amino acid substitutions in influenza virus PB2, PB1-F2, NP, and NS1 have also been assessed with regard to their contributions to enhancing viral propagation and infectivity [6–9]. However, none of these single mutations in 2009 pH1N1 IAVs sufficiently explains the pathogenicity of the 2009 pandemics. Notably, M2 protein channel activity enhanced 2009 pH1N1 IAV infectivity by protecting against premature HA cleavage and preserving membrane fusion competence. Additionally, the mutant form of NP exhibits selectively reduced NA expression, thereby indirectly increasing influenza virus fitness [10]. These findings indicate that two or more noninteracting proteins may also elicit concerted actions and that identifying sequence variations in viral or host factors that synergistically contribute to enhanced infection pathogenicity is important.

During evolution, molecules that perform similar functions tend to coevolve. In addition to physically interacting proteins, noninteracting proteins with similar functions are also expected to coevolve [11–13]. Therefore, we utilized a methodology to search for coevolution of amino acid sequence variations in 2009 pH1N1 IAV strains. In this report, we explore the synergistic effects of NP and NA substitutions that may contribute to the enhancement of influenza viral pathogenicity.

## Results

### Identification of covarying NP and NA pair RNA segments

**Fig 1:**
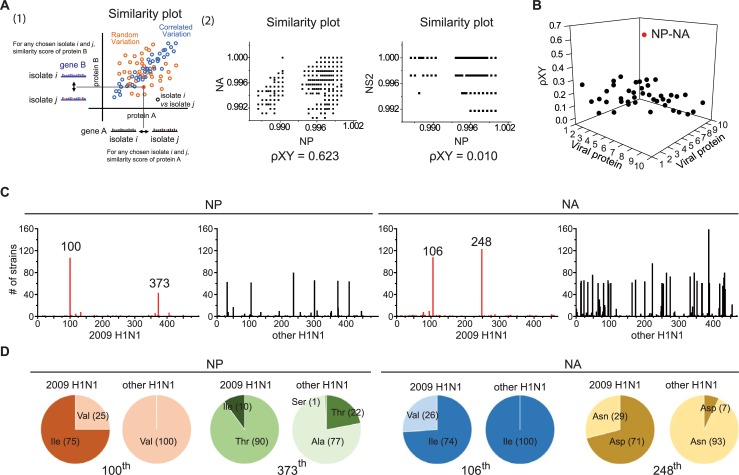
Identification of covarying NP and NA substitution pairs from the 2009 H1N1 influenza virus. (A) Schematic diagram identifying covarying viral proteins. (1) Similarity plot for any chosen gene pair (genes A and B in this case) for any chosen virus isolates *i* and *j*. Covariation of genes A and B was calculated by measuring Pearson’s correlation coefficient (ρXY) from the similarity plot. (2) Left, similarity plot for the NP and NA proteins with a ρXY = 0.623. Right, similarity plot for NP and NS2 with a ρXY = 0.01. (B) Pearson’s correlation coefficient (ρXY) for each pair of influenza viral proteins. 1: PB2, 2: PB1, 3: PA, 4: HA, 5: NP, 6: NA, 7: M1, 8: M2, 9: NS1, and 10: NS2. (C) Number of isolates with different amino acids at each position for the NP (left) and NA (right) proteins. For the 2009 pH1N1 IAV (red), 417 isolates were compared. For the other H1N1 strains (black), 267 isolates were compared. (D) Occurrence of amino acids at the 100^th^ and 373^th^ residues in NP (left) and the 106^th^ and 248^th^ residues in NA (right) in the pool of 2009 H1N1 and other H1N1 isolates.

To identify pairs of genetic traits that may have functioned to augment influenza viral pathogenicity, we examined covariance in sequence information from the viral transcripts of pH1N1 IAV isolates obtained during the 2009 pandemic season. This identification is based on the fact that genes that function together tend to covary during the course of evolution [11, 14]. In total, the sequences of 417 pH1N1 virus isolates collected in 17 countries were examined (from the Influenza Sequence & Epitope Database, ISED [15]). The number of different amino acid sequences between any chosen gene pair among the 417 isolates was counted, and the covariance of sequence variations among 10 viral proteins were compared in pairs using Pearson’s correlation analysis (Fig 1A). Among 45 total pairwise comparisons, the covariance value of the NP and NA pair was exceptionally high (Fig 1B and S1 Table). These results suggest that if one virus isolate has sequence alternations in NP proteins, then it is likely that this isolate contains a similar degree of sequence alternation in the NA protein as well.

We next assessed whether any specific amino acid substitutions co-occurred in NP and NA pairs of the 2009 pH1N1 IAVs. For this purpose, amino acid sequences from the 417 isolates were compared with those of seasonal H1N1 influenza viruses obtained from 1958 to 2008 (from ISED[15]), and variabilities at each position were separately mapped (Fig 1C). In general, relatively high variability was observed in NP and NA sequences from seasonal H1N1 influenza strains. In contrast, NP and NA sequences from isolated 2009 pH1N1 pools were relatively invariable, except for a couple of residues. For NP of the isolated 2009 pH1N1 IAVs, the 100^th^ and 373^th^ positions were highly variable. Valine (V) was observed as the 100^th^ residue of NP of the seasonal H1N1 IAVs, whereas an isoleucine (I) was present at this position in 75% of the 2009 pH1N1 isolates. For residue 373, threonine (T) was dominant NP of in isolated 2009 pH1N1 IAVs, but alanine (A) was most frequent in the seasonal H1N1 NP sequence (Fig 1D). Highly variable residues at the 106^th^ and 248^th^ positions were also observed for NA of the isolated 2009 pH1N1 IAVs: approximately 26% of the 2009 H1N1 isolates had V instead of I at the 106^th^ position of NA, and 71% had an aspartic acid (D) instead of an asparagine (N) at the 248^th^ position (Fig 1D).

### *In vitro* characterization of recombinant influenza A/WSN/1933 (H1N1) viruses carrying covarying NP and NA combination pairs

**Fig 2:**
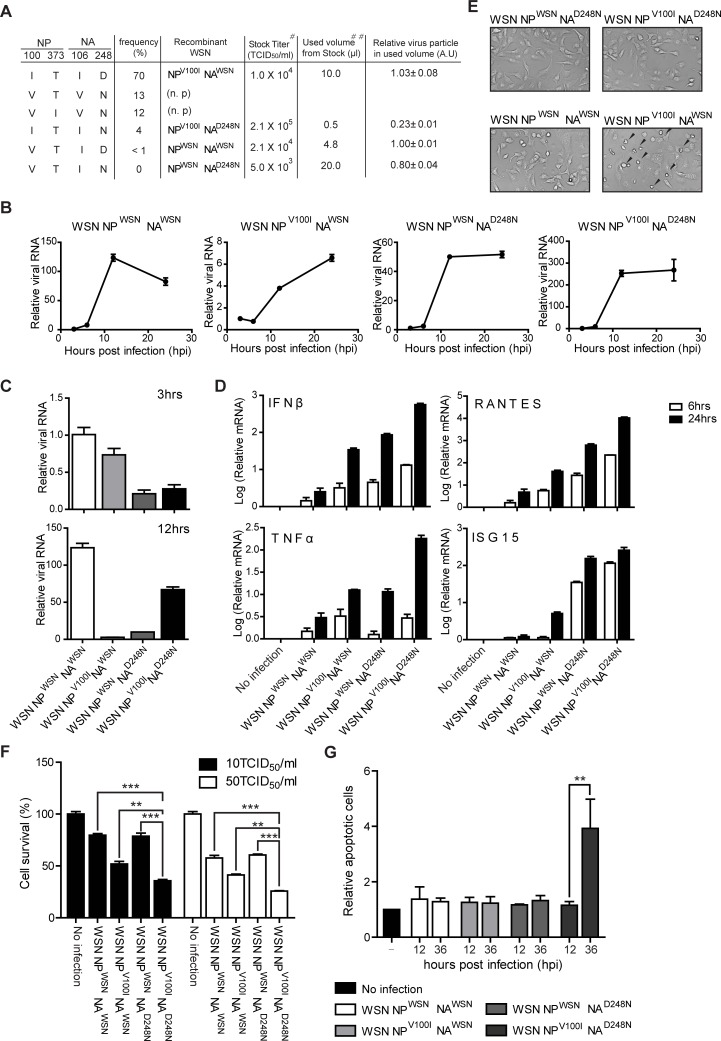
Recombinant WSN carrying the NP^V100I^ NA^D248N^ pair exhibited enhanced pathogenicity *in vitro*. (A) Recombinant WSN was generated with the designated substitutions. Frequency (%) refers to the occurrence among the 417 2009 H1N1 isolates. # the stock titer (TCID_50_/ml) was assessed in A549 cells for 48 hours, ## the used volume of influenza virus stock for 1 ml of 10TCID_50_/ml, n.p. not produced. (B and C) A549 cells were infected with the indicated recombinant WSN viruses (10 TCID_50_/ml, 0.01 MOI) for the indicated times. Influenza HA viral RNAs were analyzed via quantitative real-time PCR (qRT-PCR). (B) The intracellular amounts of each virus at 6, 12, 24 hours postinfection was measured. The relative values to the viral load at 3 hours postinfection for each virus. (C) Same data as (B), but relative value to the NP^WSN^-NA^WSN^ influenza virus (3 hours postinfection) (D) A549 cells were infected with the indicated recombinant viruses (10 TCID_50_/ml, 0.01 MOI) for 6 or 24 hours. The expression levels of type I interferons, TNFα, RANTES, and interferon stimulated gene 15 (ISG15) were analyzed by quantitative real-time PCR (qRT-PCR). (E) A549 cells were infected with the indicated recombinant viruses (10 TCID_50_/ml, 0.01 MOI) for 48 hours, and the alterations in cell morphology were evaluated under bright-field microscopy. (F) A549 cells were infected with different doses of recombinant viruses for 48 hours. Cell survival was determined by staining with 2% crystal violet. The bar graphs reflect the quantitation of crystal violet staining. (G) A549 cell death was measured by Annexin V and PI double staining after 12 or 36 hours of infection (10 TCID_50_/ml, 0.01 MOI). Each bar graph represents data obtained from three independent experiments. The mean and standard error were analyzed by one-way ANOVA followed by the Newman-Keuls posttest in GraphPad Prism 5. ns, not significant; **p<0.01; and ***p<0.001.

We classified the NP and NA sequence information at the 100^th^, 373^rd^, 106^th^, and 248^th^ positions of the 2009 pH1N1 isolates (Fig 2A). Many 2009 pH1N1 isolates had NP^100I,373T^/NA^106I,248D^ (I-T/I-D); a minor portion had NP^100V,373T^/NA^106V,248N^ (V-T/V-N), NP^100V,373I^/NA^106V,248N^ (V-I/V-N), NP^100I,373T^/NA^106I,248N^ (I-T/I-N), and NP^100V,373T^/NA^106I,248D^ (V-T/I-D), in that order. To investigate the functional characteristics of these specific amino acid combinations of the NA and NP variants observed among the 2009 pH1N1 isolates, we generated recombinant viruses carrying these substitutions at each NP and NA position in the laboratory-adapted influenza A/WSN/1933(H1N1) background strain. However, recombinant viruses carrying NA^106V,248N^ were not produced, even though NA proteins were expressed (Fig 2A and S1 and S2 Figs), indicating that these NA amino acid pairs might result in a defect in release processes. Recombinant WSN virus with the remaining combinations, namely, I-T/I-D, I-T/I-N, and V-T/I-D, were successfully produced. As the parental WSN strain expresses V-T/I-D and the generated recombinant viruses have the same amino acids at the 373^th^ position of NP and the 106^th^ position of NA, we analyzed the combined effect of NA and NP using recombinant viruses carrying a single substitution at the 100^th^ position of NP (NP^V100I^ NA^WSN^) or double substitutions at the 100^th^ position of NP and the 248^th^ position of NA (NP^V100I^ NA^D248N^). For comparison, a recombinant WSN virus carrying NP^WSN^ NA^D248N^ was also generated (Fig 2A and S2 Fig).

First, we measured the virus titer (TCID_50_/ml) of the recombinant WSN viruses and the relative number of virus particle present based on M gene quantitation. To evaluate the replication kinetics of the recombinant WSN viruses, A549 cells were infected with the same amounts of recombinant WSN (10 TCID_50_/ml, 0.01MOI), and intracellular viral loads were measured at 3, 6, 12, and 24 hours postinfection. The relative virus particle numbers used in this experiment were similar in range, except for WSN viruses with the NP^V100I^ and NA^D248N^ mutations (Fig 2A), which were approximately one-fifth lower. To compare the differential kinetics of viral growth, viral loads at 6, 12, and 24 hours postinfection for each virus were separately normalized to the viral load at 3 hours postinfection for each virus. Although relative amount of intracellular viral genome was lower with NP^V100I^ NA^D248N^ influenza virus infection compared to NP^WSN^ NA^WSN^ influenza virus infection, WSN carrying the NP^V100I^ NA^D248N^ combination exhibited a high replication rate during the 24-hour period compared to the parental or other recombinant WSN viruses (Fig 2B and 2C). This result indicates that the presence of NA^D248N^ in addition to NP^V100I^ significantly enhanced relative infectivity compared to that of the parental WSN virus. Although infection with either NP^WSN^ NA^D248N^ WSN or NP^V100I^ NA^WSN^ WSN resulted in relatively higher levels of type I interferons (type I IFNs) and inflammatory cytokines, infection with NP^V100I^ NA^D248N^ WSN led to enormously high levels of cytokines, which cannot be explained by the additive effect of the dual NP and NA substitutions (Fig 2D). We postulate that cytokine production might be indirectly enhanced by the increased viral production of NP^V100I^ NA^D248N^ influenza viruses. In addition, the recombinant NP^V100I^ NA^D248N^ WSN virus was also more toxic to host cells than was the parental or other single-mutant WSN virus (Fig 2E–2G). These findings indicate that a direct or indirect functional relationship may exist between NP^V100I^ and NA^D248N^, possibly promoting influenza viral pathogenicity.

### Recombinant NP^V100I^ NA^D248N^ WSN exhibits enhanced pathogenicity *in vivo*

**Fig 3:**
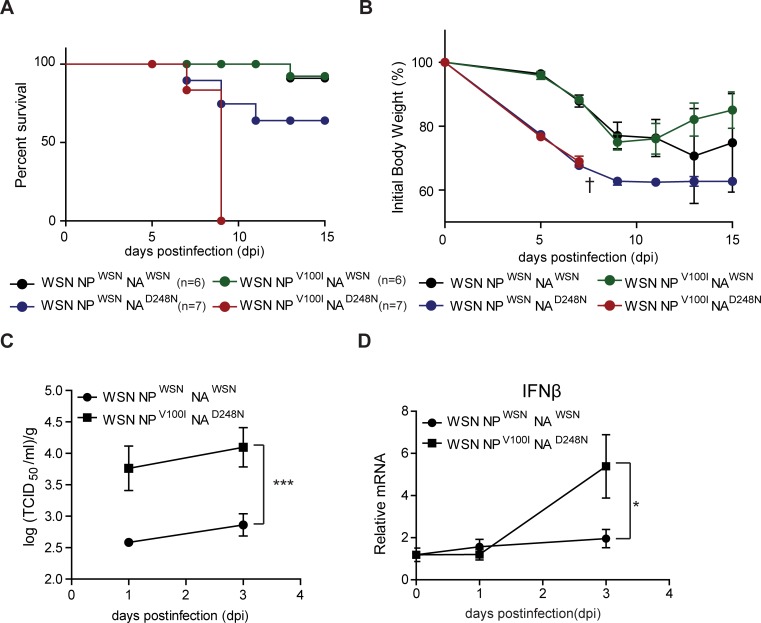
Recombinant WSN carrying the NP^V100I^ NA^D248N^ pair exhibited enhanced pathogenicity *in vivo*. (A and B) BALB/c mice were intranasally infected with the recombinant WSN virus (2000 TCID_50_/ml, 100 μl), and body weight and mortality were measured. † indicates the time point when all of the NP^V100I^ NA^D248N^ WSN-infected mice were dead. (C and D) Lung homogenates from infected mice were prepared at the indicated time points. (C) Viral titers were measured in A549 cells, and (D) IFNβ expression was determined by qRT-PCR. The mean and standard error obtained from six mice were analyzed by one-way ANOVA followed by the Newman-Keuls posttest in GraphPad Prism 5. * p<0.05 and ***p<0.001.

We next evaluated the pathogenic effect of the NP^V100I^ NA^D248N^ WSN virus in BALB/c mice. Mice were intranasally infected with recombinant WSN influenza viruses (100 μl, 2000 TCID_50_/ml), and their survival rates were observed over a two-week period. For comparison, groups of mice were also infected with the NP^V100I^ NA^WSN^ or NP^WSN^ NA^D248N^ WSN virus. Infection with the NP^V100I^ NA^D248N^ WSN virus caused high mortality, with every mouse tested dying by 9 days postinfection (d.p.i.) (Fig 3A). In contrast, greatly reduced mortality was found with infection of either NP^V100I^ NA^WSN^ or parental WSN, with over 40% of NP^WSN^ NA^D248N^ WSN-infected mice being dead at 15 d.p.i. (Fig 3A). Mice infected with either NP^V100I^ NA^WSN^ or the parental WSN experienced weight loss but recovered, though infection with NP^WSN^ NA^D248N^ WSN resulted in nearly 30% weight loss at 15 d.p.i. (Fig 3B).

To better understand the pathogenicity of NP^V100I^ NA^D248N^ WSN, we next investigated the kinetics of viral replication and IFNβ production at an early time in the lungs of infected mice to evaluate viral replication alone and ignore factors influencing mouse survival. Viral titers obtained from the lungs of mice infected with NP^V100I^ NA^D248N^ recombinant WSN were significantly higher than those of mice infected with the parental WSN virus (Fig 3C). Furthermore, mice infected with the recombinant NP^V100I^ NA^D248N^ WSN virus were unable to clear the virus and produced higher levels of IFNβ than did mice infected with the parental WSN virus (Fig 3D). Altogether, these data strongly indicate that in contrast to control or single-mutant viruses, the NP^V100I^ NA^D248N^ WSN virus exhibits strong pathogenicity *in vivo*.

### The NP^V100I^ and NA^D248N^ combination enhances the binding of viral RNA by NP

**Fig 4:**
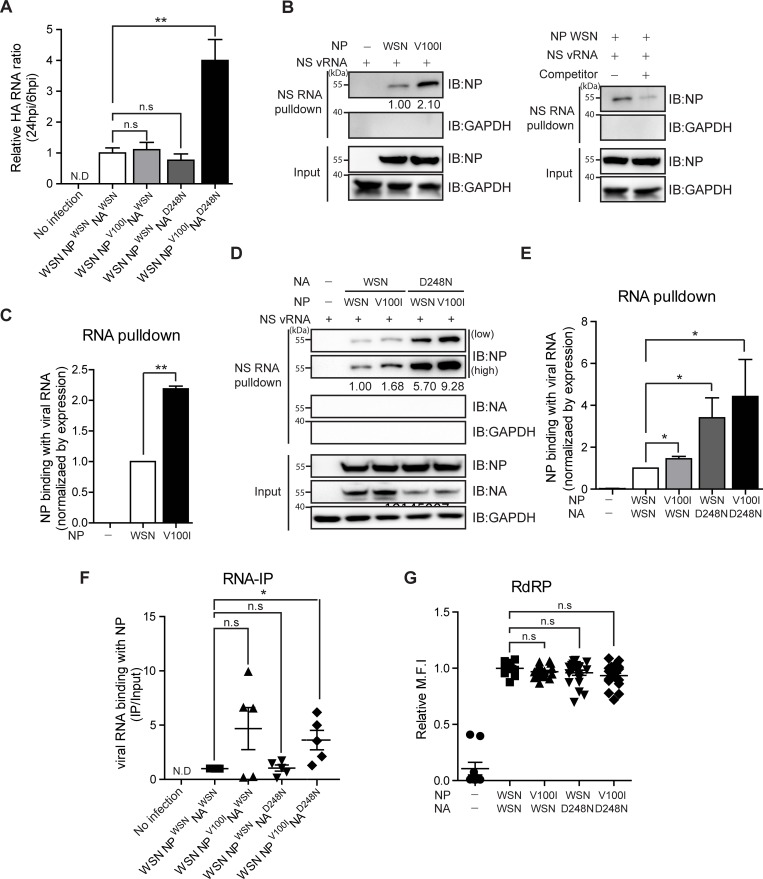
NP-mediated RNA binding is significantly increased with covarying NP^V100I^ and NA^D248N^. (A) A549 cells were infected with the indicated recombinant WSN virus (10 TCID_50_/ml, 0.01 MOI), and intracellular viral RNA levels were measured at 6 or 24 hours postinfection (h.p.i.). Accumulation of the viral genome was measured by calculating the ratio between the amount of intracellular viral RNA at 24 h.p.i. versus that at 6 h.p.i. Each bar represents three independent experiments. (B and D) A biotin-labeled NS vRNA pulldown assay was performed using 293T cell lysates transfected with the indicated plasmids (48 hours). Nonlabelled NS vRNA was used as a competitor. (C and E) Western blotting data represent one result from three or four repeated independent experiments. (F) RNA immunoprecipitation of NP proteins was performed using A549 cells infected with the indicated WSN influenza A viruses (10 TCID_50_/ml, 0.01 MOI, 24 hours). Relative amounts of NP-bound vRNAs were analyzed by qRT-PCR. (G) 293T cells were transfected with expression plasmids carrying PB2, PB1, and PA along with the designated NP and NA. To measure RdRP activity, equal amounts of the NS-GFP reporter plasmid were cotransfected. The mean fluorescence intensities (MFI) of GFP-expressing cells measured by FACS are shown. (C and D) The blots shown are cropped from different parts of the same membrane after stripping. The dot graph was obtained from five independent experiments, and each individual experiment was normalized to the control. The mean and standard error were analyzed by one-way ANOVA followed by the Newman-Keuls posttest in GraphPad Prism 5. ns, not significant; *p<0.05; and **p<0.01.

In contrast to parental or single NP^V100I^ or NA^D248N^ mutant WSN viruses, the replication rate of NP^V100I^ NA^D248N^ WSN was strongly increased (Fig 2A). To exclude possible variations at the entry steps of infection, we measured accumulation of the intracellular viral genome at 24 hours relative to the intracellular viral genome at 6 hours postinfection (Fig 4A). In this case, NP^V100I^ NA^D248N^ WSN again exhibited the highest viral genome accumulation, confirming that this double mutant virus shows enhanced viral production compared to that of the parental or single-mutant virus. These results suggest that NP^V100I^ and NA^D248N^ might function together to increase the overall fitness of infectious influenza viruses.

To address the molecular mechanism of functional synergy between the NP^V100I^ and NA^D248N^ proteins, we first assessed whether physical interactions between the NP and NA proteins are affected by these substitutions. To this end, mammalian expression plasmids carrying NP^WSN^, NP^V100I^, NA^WSN^, or NA^D248N^ were generated separately, and their interactions were assessed in 293T cells. However, no physical interactions between these proteins were observed (S3 Fig). Therefore, we next hypothesized that the function of one protein might be indirectly aided by the other protein in the presence of the designated substitution. NP binds to viral RNA and is a component of the viral ribonucleoprotein (vRNP) complex [16, 17], and V100I is located near the RNA-binding domain [18] (S4 Fig). We first examined whether the RNA-binding potential of NP is altered by this substitution using an RNA pulldown assay with biotin conjugated to WSN NS vRNA. Cell lysates from 293T cells transfected with NP^WSN^ or NP^V100I^ expression plasmids were incubated with labeled WSN NS vRNA, and the RNA-bound form of the NP protein was visualized by an immunoblot assay. In contrast to weak NP^WSN^ binding, enhanced vRNA binding to NP^V100I^ was detected (Fig 4B and 4C). Interestingly, vRNA binding by the NP protein, either NP^WSN^ or NP^V100I^, was further increased when NA^D248N^ was coexpressed (Fig 4D and 4E), indicating that NA^D248N^ is able to aid NP protein-vRNA interactions. Next, an RNA-IP assay was performed in which total lysates of A549 cells infected with various forms of recombinant WSN viruses were incubated with an anti-NP antibody followed by analysis of the bound form of viral RNAs. Although NP^V100^ protein binding to viral RNA appeared to increase, the results were not significant. However, NP^V100I^ binding to viral RNA significantly increased when was NA^D248N^ also present (Fig 4F). These data, along with those of the transfection experiments, confirm that the NP^V100I^ interaction with viral RNA is strengthened in the presence of NA^D248N^.

To better understand the altered function of NP proteins by substitution or coexpression with NA^D248N^, we examined viral genome replication because NP has been reported to function in viral genome replication steps along with other PB1/PB2/PA RNA polymerase complex components [16]. 293T cells were cotransfected with NP^WSN^, NP^V100I^, NA^WSN^, or NA^D248N^ along with an eGFP reporter plasmid carrying NS vRNA, and the viral RNA-dependent RNA polymerase (RdRP) activity of NP was assessed by measuring the mean fluorescent intensity of GFP (Fig 4G). Under all conditions tested, the RdRP activity of the NP protein was not significantly altered by single-amino acid substitutions at the 100^th^ position or in combination with NA^D248N^ expression.

### The NA^D248N^ protein is recruited to the lipid raft compartment

**Fig 5:**
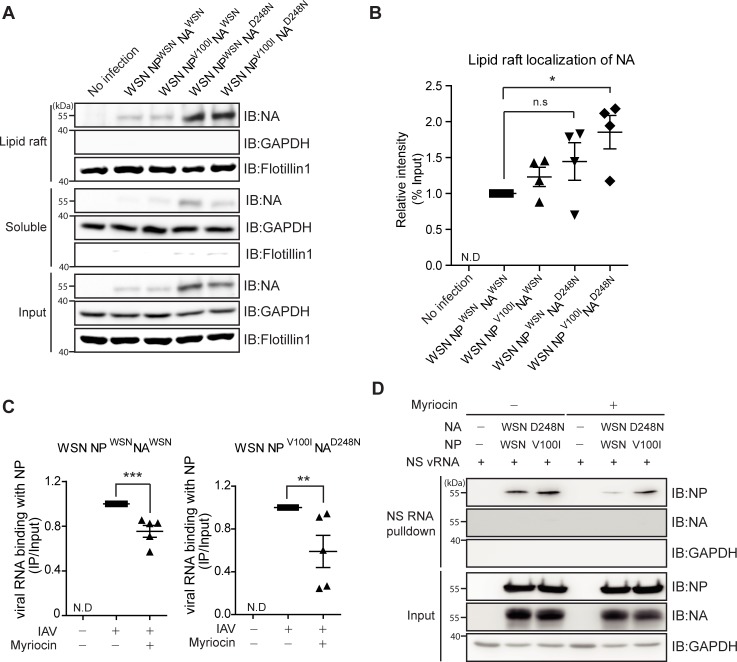
NA trafficking to the lipid raft compartment was enhanced by the NA^D248N^ substitution. (A and B) A549 cells were infected with the indicated NP- or NA-containing recombinant WSN virus (10 TCID_50_/ml, 0.01 MOI, 48 hours), and the lipid raft-enriched membrane compartment was extracted in the TX-100 insoluble fraction. Flottilin 1, lipid raft marker and GAPDH, soluble fraction marker. The graph was obtained from four independent experiments, and each individual experiment was normalized to the control. (C) A549 cells were pretreated with myriocin (5 μM for 72 hours) or DMSO and infected with the indicated recombinant WSN (10 TCID_50_/ml) influenza A virus for 12 hours. RNA IPs were performed. The graph was obtained from five independent experiments, and each individual experiment was normalized to the control. (D) A biotin-labeled NS vRNA pulldown assay was performed using lysates of myriocin (5 μM for 72 hours)-pretreated 293T cells transfected with the indicated plasmids. (A and D) The blots shown are cropped from different parts of the same membrane after stripping. The mean and standard error were analyzed by one-way ANOVA followed by the Newman-Keuls posttest in GraphPad Prism 5. ns, not significant; *p<0.05, **p<0.01, and ***p<0.001.

Herein, we provide evidence that vRNA-NP assembly as well as viral production are synergistically enhanced when both NP^V100I^ and NA^D248N^ are present, indicating that the gain of function effect by NA^D248N^ substitution influences the function of NP^V100I^, ultimately promoting viral production. During assembly of newly synthesized influenza viral proteins and vRNPs, the HA and NA proteins are independently targeted toward lipid rafts [19, 20], which is also required for the apical targeting of vRNPs [21]. In transfected 293T cells, a large amount of NA^D248N^ protein was observed in cellular compartments containing lipid raft markers (S5 Fig). Therefore, we hypothesized that intracellular trafficking of the vRNP complex to lipid rafts is affected by NA^D248N^, which may explain the enhanced viral production of recombinant NP^V100I^ NA^D248N^ WSN. To test this hypothesis, we measured the lipid raft trafficking of NA in recombinant virus-infected A549 cells. Similar to plasmid transfection, more NA protein was observed in the lipid raft compartment of cells infected with NA^D248N^ WSN (Fig 5A). In this case, the input expression of NA was also increased after NA^D248N^ WSN infection. Furthermore, statistically significant enhancement of NA localization in the lipid raft compartment was observed in cells infected with NP^V100I^ NA^D248N^ WSN virus (Fig 5B). These data suggested that NA^D248N^ mutation causes increased NA levels, which could account for the enhanced NA lipid raft association.

To determine the functional relationship between enriched NA at lipid rafts and the enhanced RNA-binding of NP, cells were treated with a myriocin, a serine palmitoyltransferase inhibitor that depletes sphingolipid synthesis and inhibits lipid raft formation, and then NP protein binding to RNA was measured. Depletion of the lipid raft compartment in myriocin-treated cells was confirmed by observing cholesterol reduction at the plasma membrane using FITC-conjugated cholera toxin-B, and we found that NA association with lipid rafts was reduced by myriocin treatment (S6 Fig). Cells were infected with either NP^WSN^ NA^WSN^ WSN or NP^V100I^ NA^D248N^ WSN virus, and NP-mediated viral RNA binding was compared in the lipid raft-depleted condition. In myriocin-treated cells, NP binding to viral RNA was significantly reduced with both NP^WSN^ NA^WSN^ WSN and NP^V100I^ NA^D248N^ WSN infection (Fig 5C). In addition to virus infection, reduced NP binding to vRNA was observed in myriocin-treated, expression-plasmid-transfected cells (Fig 5D). Taken together, these data indicate that enhanced accumulation of NA at lipid rafts might contribute to the enhanced RNA binding of NP, which does explain the synergistic effect of NP^V100I^ and NA^D248N^ in promoting influenza virus pathogenicity.

## Discussion

**Fig 6:**
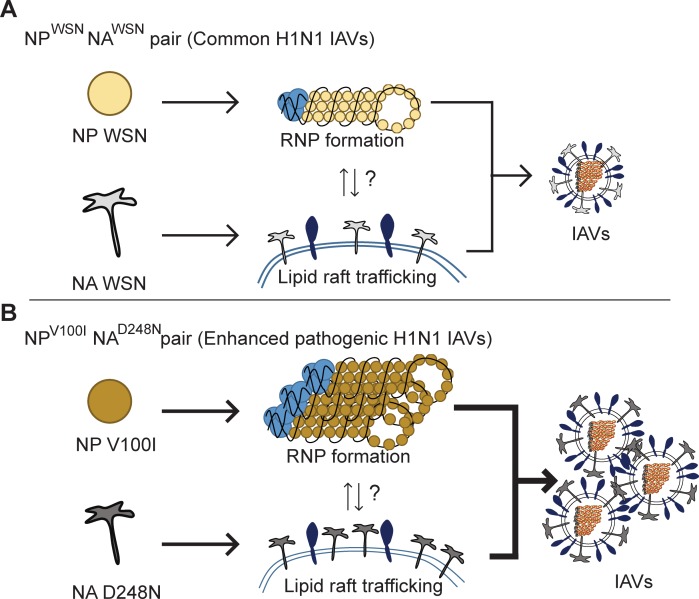
Working model of the functional synergy between the covarying NP^V100I^ and NA^D248N^ pair in 2009 pH1N1 IAVs. (A) Normal conditions for WSN influenza A virus production. (B) For recombinant WSN, NP^V100I^ showed increased vRNA binding, which enhanced viral RNP assembly. Simultaneously, NA^D248N^ exhibited increased recruitment to the lipid raft compartment, which promoted virus particle release. Although covarying NP^V100I^ and NA^D248N^ proteins do not physically interact, functional synergy leads to augmented production of the 2009 H1N1 influenza virus.

In this report, we demonstrate that functional synergy between the covarying NP^V100I^ and NA^D248N^ mutation pairs of the 2009 pH1N1 IAV explains the enhanced pathogenicity of this influenza virus. A single-amino acid substitution at the 100^th^ residue of the NP protein increased its vRNA binding potential, which was significantly enhanced when the NA^D248N^ protein was present. The ability of the NA protein to be recruited to the lipid raft compartment, which directly influences viral particle release, was also augmented with a single-amino acid change at the 248^th^ residue of the NA protein. Therefore, our data indicate that the covarying NP^V100I^ and NA^D248N^ pair functions to boost viral component assembly and virus particle release, which may explain the increased production of influenza virus (Fig 6).

We utilized amino acid sequence information from 2009 pH1N1 IAV isolates to search for covarying protein pairs based on the assumption that proteins that function together have a high probability of coevolution. Although we interpreted high covariation as a sign of a functional relationship, covariation can be affected by many factors in addition to the pressure to coevolve, and geographic distance, evolutionary distance between viruses, or gene lengths can influence our results. We consider two possible scenarios if geographic distance dominates impacts on covariation: 1) covariation among all genes should be very low, as approximately 70% (291/417) of examined viral sequences were obtained from a single country (USA); or 2) the total covariation score should be high because correlation according to distance is very significant, even among viruses from the USA. In general, our covariation analysis revealed relatively low correlation coefficients (between -0.104 and 0.253), except for the NP-NA pair (0.623). The length of a sequence is another potential factor that can affect the probability of random variation. To address this issue, the similarity value was normalized to the total number of sequences. Analysis of the covariation results indicated no correlation with sequence length: the longest genes (PB2, PB1) did not show the highest correlation, and the shortest genes (NS1, NS2, M2) did not show in relatively small correlations among themselves or with other genes (S1 Table). Based on this interpretation, we postulated that the influence of sequence length on covariation was minimal, if at all.

Physically interacting proteins coevolve to complement structural changes at the interaction surface. When coevolutionary selection pressure is applied to pairs of interacting proteins, amino acid substitutions in one protein often affect the rates of substitutions in interacting partners. By measuring correlating variability of amino acid sites among proteins, structural as well as phylogenetic coevolution can be assessed [12, 14]. Although most molecular coevolution studies have focused on interacting proteins, accumulating evidence indicates that noninteracting protein pairs with similar functions also coevolve. In fact, on a proteome-wide scale, analyses of evolutionary rate covariation (ERC) along with physically and genetically interacting proteins and noninteracting but functionally related proteins have revealed significant ERC [13]. Although biases and limitations are present, diverse analytical tools for measuring amino acid covariation have been developed and applied, leading to breakthrough studies for predicting protein structure and interactomes [12, 22]

In general, measuring covariation is extremely useful when employing a molecular approach to understand virus pathogenicity. For example, covariation between mutations in the variable site of the V3 loop of the human immunodeficiency virus type 1 envelope protein has been assessed to identify significant covarying mutations with structural or functional relationships [23]. In addition, covariation of eight amino acids in the capsid protein of Hibiscus chlorotic ringspot virus (HCRSV) has been identified as the genetic change responsible for the loss of virulence after serial passaging [24]. The amino acid covariance of the hepatitis C virus (HCV) viral coding region in infected patients has been measured before treatment to link covarying positions with differential responses to therapy [25], and genome-wide analysis of HCV covariance networks has been applied to identify amino acid pairs predicted to contribute to therapy failure as well as hub positions with antiviral target potential. In contrast to specific amino acid site-wise level comparison analysis, we measured the similarity between entire sequences, which distinguishes our approach from other more typical methods. In this study, we examined the amino acid covariance of viral proteins of the 2009 pH1N1 influenza virus to identify genetic factors contributing to the pathogenicity of the 2009 pandemic. A total of 417 whole-genome sequences from independent influenza virus isolates were compared to assess covariance, and covarying pairs of the influenza viral proteins NA and NP were identified. However, our analysis has some potential shortcomings. Among the 417 examined isolates, approximately 70% of the information was obtained from a single country (USA, low geographic distance), which suggests that our data analysis of predominant amino acid sequences at variable residues in NP or NA might be affected by limited sequence information.

We demonstrate that WSN with the NP^V100I^ NA^D248N^ pair was highly pathogenic. However, a low percentage of viruses with the NP^V100I^ NA^D248N^ pair were found among 2009 pH1N1 IAVs, with a naturally occurring frequency of only 4% (Fig 2A). Such an apparent contradiction can also be explained by considering the relatively mild pathogenicity of 2009 pandemic influenza. We postulate that highly pathogenic 2009 pH1N1 IAVs may have failed to adapt among the host population but that rather mildly pathogenic 2009 pH1N1 IAVs were well adapted and dominant. It is likely that the most pathogenic virus eliminated its host too rapidly to propagate, resulting in a small population of infected hosts. Alternatively, the pathogenic NP^V100I^ and NA^D248N^ combination may have caused a phenotype that prevented spread, yet we cannot present a possible mechanism that supports this hypothesis.

Influenza NP is a single-stranded RNA (ssRNA)-binding protein that encapsidates the viral RNA genome to form a ribonucleoprotein particle. NP also interacts with several viral and host proteins to elicit multiple functions during the virus replication cycle of influenza infection [18]. NP participates in viral RNA transcription processes through interactions with the RdRP complex and viral packaging; it also interacts with host factors, such as the human chaperone heat shock protein 40, to modulate PKR activation or inhibit IRF3 activation [26]. Both the sequence comparison and reverse genetics approaches applied to identify substitutions responsible for the pathogenicity observed in the 2009 pH1N1 influenza outbreak have mainly been concentrated on the membrane proteins HA and NA, with a limited number of studies reporting the role of NP. In this study, we observed that the single-amino acid substitution of V to I at the 100^th^ position near the RNA-binding domain enhanced NP-mediated RNA-binding ability but not RdRP activity. Interestingly, the increased RNA binding, or rather RNP formation, resulted in enhanced virus production, especially when a covarying amino acid substitution was present in the NA protein, affecting lipid raft recruitment.

During the final stage of virus assembly, the packaged RNA-protein complex associated with the intracellular membrane translocates to the apical side of the lipid raft-rich plasma membrane, and this is a critical step for virus release [27]. We observed that a transfected NA (D248N) protein or recombinant virus with an NA (D248N) substitution was recruited to the lipid raft compartment more frequently than was the control protein or virus, respectively. The D248 residue is located in the extracellular domain of the NA protein, and no reports thus far suggest a role for lipid raft trafficking within this region [19, 20]. Therefore, we hypothesize that physical interactions between NA and host factors within the lumen of vesicular endosomal organelles, such as the trans-Golgi apparatus, might be disrupted by this amino acid substitution. Interestingly, galectin-1, which functions to sort apical glycoproteins or their apical trafficking, reportedly interacts with influenza virus envelope proteins and controls viral infectivity [28, 29]. Thus, investigating whether the identified substitutions in the NA protein alter its interaction with host trafficking proteins is warranted.

Covariation analysis of 2009 pH1N1 influenza sequence information revealed covarying viral proteins and specific amino acid substitutions that synergistically influence the pathogenicity of infection. Although no obvious physical interactions were observed, we experimentally demonstrated that these covarying pairs of genetic factors function together to enhance viral production. Based on these results, we propose that covariation analysis is needed to screen genetic factors that do not interact but instead function together.

## Materials and methods

### Identification of covarying gene pairs

Similarity scores of amino acid sequences were calculated for every pair of isolates for each influenza viral protein, and covariations of viral proteins were analyzed by Pearson’s correlation coefficient (*ρ*). The amino acid sequences of influenza virus strains were aligned and compared using parsing methods in Biopython. The similarity score for a viral protein was calculated as a ratio of matched sequences between two strains among total amino acid sequences. Similarity scores were calculated for all possible combinatory pairs of pandemic strains per influenza protein with general programming using MATLAB.

$$Similarity\;(strain\;i,strain\;j)=\frac{Number\;of\;matched\;residues\;between\;strain\;i\;and\;strain\;j}{Total\;number\;of\;residues}$$

The degrees of correlated mutations between viral proteins were analyzed by Pearson’s correlation coefficients (*ρ*) of similarity scores of same-strain pairs using the standard function *corr* in MATLAB.

$$\rho_{proteinA,proteinB}=\frac{Covariance\;(similarity\;score\;of\;protein\;A,similarity\;score\;of\;protein\;B)\;}{\sigma_{\left(similarity\;of\;protein\;A\right)}∙\sigma_{\left(similarity\;of\;protein\;B\right)}}$$

To identify and quantify degree of variability at each amino acid position in a protein, the RNA sequence was translated into the amino acid sequence and aligned using standard procedures in Biopython; analysis by general programming was performed using MATLAB. In addition, the number of strains that had different amino acids compared to the most frequent amino acid at each position was counted.

The sequence information for the 417 2009 pH1N1 IAVs and the sequence information for the 267 1948 to 2008 seasonal H1N1 IAVs were obtained from the ISED on December 2011.

### Cell culture and reagents

Adenocarcinomic human alveolar basal epithelial cells (A549, ATCC) and human embryonic kidney cells with SV40 T-antigen (293T, ATCC) were maintained in Dulbecco’s Modified Eagle’s Medium (DMEM, Gibco) supplemented with 10% fetal bovine serum (FBS) (HyClone) and 1% penicillin/streptomycin (Invitrogen). Myriocin (Sigma) was dissolved in dimethyl sulfoxide (DMSO, Sigma), and DMSO was used as a control.

### *In vitro* virus production and virus infection

Recombinant WSN influenza A virus was generated using an *in vitro* virus production method [30]. A total of 12 plasmids, pCAGGS WSN PB2, pCAGGS WSN PB1, pCAGGS WSN PA, pCAGGS WSN NP 0/14, pHH21 polI WSN PB2, pHH21 polI WSN PB1, pHH21 polI WSN PA, pHH21 polI WSN NP, pHH21 polI WSN HA, pHH21 polI WSN NA, pHH21 polI WSN NS, and pHH21 polI WSN M (kindly provided by Drs. Kawaoka and Neumann, Madison-Wisconsin Univ., USA), were cotransfected into 293T cells using the calcium phosphate method. For NA or NP substitutions, the pHH21 polI WSN NP or pHH21 polI WSN NA expression plasmids with designated changes were generated. Twelve hours after transfection, cells were washed with PBS and further incubated in DMEM containing 10% FBS for 48 hours. The supernatants were harvested, and the recombinant viruses were isolated by sucrose density gradient (30%/60%) centrifugation. Recombinant viruses were then propagated in chicken embryonated eggs for 48 hours, and the TCID_50_/ml values of the recombinant viruses in A549 cells were calculated.

Recombinant WSN influenza A virus was infected into A549 cells at the indicated titer (TCID_50_/ml). Three hours later, the infected cells were washed with PBS to remove semi-infectious nonbound virus and incubated for the indicated time.

### Mouse experiments

BALB/cOlaHsd (BALB/c) mice were obtained from Harlan Olac (Bicester, UK). After being intraperitoneally (i.p.) anesthetized with ketamine (100 mg/kg; Yuhan, Korea) and xylazine hydrochloride (10 mg/kg; Bayer, Belgium) in PBS, the 6 or 7 mice from each group were intranasally (i.n.) injected with WSN influenza A virus (2000 TCID_50_/ml, 100μl). This experiment was carried out for 15 days, and mice body weight and mortality were checked and monitored every 2 days from the 5th day after the initial virus infection (n = 27). To assess mortality, mice that lost more than 35% of their initial body weight were immediately euthanized by CO_2_ inhalation or cervical dislocation (n = 14). At the end of the experiment, the mice that survived were also euthanized the same way (n = 13). For preparation of the lungs of infected mice, lungs were extracted after PBS perfusion and kept frozen at -80°C. The isolated lungs were homogenized in PBS, and the supernatants were collected by centrifugation at 13,200 rpm for 30 min at 4°C. To measure titers, A549 cells were treated with homogenate supernatants from the virus-infected lungs. For mRNA expression, total RNA was prepared from 200 μl of tissue homogenate.

### Ethics statement

The production of recombinant WSN IAVs was performed in compliance with the guidelines established by the Genetic Recombination Experiment Policy of the Institutional Biosafety Committee (IBC) at POSTECH under approval number PIBC-008.

Mouse experimental procedures were approved by the Institutional Animal Care and Use Committee (IACUC) at POSTECH under the standard IACUC guidelines (registration number: 11-1543061-000268-14) of the Animal and Plant Quarantine Agency (APQA) and Ministry of Food and Drug Safety (MFDS) of Korea with permit number POSTECH-2014-0002 (Experimental influenza infection). All mouse experiments were performed in a Biosafety level 2 facility that was accredited by the Korea Centers for Disease Control & Prevention (KCDC). For the data presented, 27 mice were used, of which 13 were euthanized at end of experiment and 14 in anticipation of reaching the humane endpoint. Dead mice were not found.

### Crystal violet assay & apoptosis analysis

A549 cells in a 24-well plate (2 × 10^4^ cells/well) were infected with recombinant WSN IAV. After the indicated time, the cells were washed with PBS and fixed with 4% paraformaldehyde (Sigma) for 10 min at room temperature (RT). After staining with 2% crystal violet (Sigma) (dissolved in 50% ethanol), the dye crystals were dissolved in a 1% SDS solution at 37°C for 10 min and analyzed using an EZ Read 400 instrument (Biochrom, UK).

For measuring apoptosis, A549 cells (5 ×10^5^) were infected with the indicated recombinant WSN IAV for 12 or 36 hours. The cells were stained with Annexin V (BD bioscience) and propidium iodide (Sigma) in 10 mM HEPES (pH 7.4), 140 mM NaCl, and 2.5 mM CaCl_2_ for 20 min at RT and analyzed using a FACS caliber flow cytometer (BD Biosciences).

### Immunoblot analysis

Cells were lysed in lysis buffer (150 mM NaCl, 25 mM Tris-HCl (pH 7.4), 1% Triton X-100, 0.5% deoxycholic acid, and 0.1% SDS) supplemented with protease inhibitors (1 mM DTT, 2 μg/ml pepstatin, 1.1 mg/ml phenylmethanesulfonyl fluoride, 5 μg/ml aprotinin, 5 μg/ml leupeptin, and 1 mM benzamidine). Total cell lysates were separated by SDS-PAGE, and proteins were transferred to nitrocellulose membranes (Whatman). For immunoblotting, the membranes were blocked in a 6.5% skim milk solution and probed first with primary antibodies and then with horseradish peroxidase (HRP)-conjugated secondary antibodies. Signals were visualized using the LAS4000 image reader (Fuji Film) after ECL treatment (Pierce). The following primary antibodies were used: anti-NP (1:2,000, Abcam, ab128193), anti-WSN NA (1:2,000, Pierce, PA5-32238), anti-Flotillin1 (1:1,000, Santa-Cruz Biotechnology, sc-25506), anti-M2 (1:1,000, Santa-Cruz Biotechnology, sc-32238), anti-Lamin B2 (1:1,000, Santa-Cruz Biotechnology, sc-6216), and anti-GAPDH (1:25,000, Chemcon, MAB374). The secondary antibodies used for these experiments were as follows: goat anti-mouse HRP-conjugated IgG (1:50,000, Pierce, 1858413), goat anti-rabbit HRP-conjugated IgG (1:5,000, Pierce, 1858415), and donkey anti-goat HRP-conjugated IgG (1:5,000, Santa-Cruz Biotechnology, sc-2020). To measure viral release, equal amounts of cell culture media were loaded into a dot blotter (Biometra, Whatman company) followed by immunoblotting using the anti-M2 antibody.

### RdRP activity analysis

293T cells were transfected with the pCAGGS WSN PB2, pCAGGS WSN PB1, pCAGGS WSN PA, pCAGGS WSN NP 0/14 (WT or V100I), pCAGGS and WSN NA (WT or D248N) expression vectors along with the pHH21 WSN NS-GFP reporter plasmid. Thirty-six hours after transfection, GFP-expressing cells were analyzed using a FACS caliber flow cytometer.

### Total RNA isolation and real-time quantitative PCR

Total RNA was extracted using RNAiso plus (Takara Bio), and 1 μg was reverse transcribed with ImProm-II Reverse Transcriptase (Promega) using random primers (Promega). PCR analysis of the cDNA was performed using a StepOne Plus Real-Time PCR machine (Applied Biosystems). The 18s rRNA or hGAPDH gene was used as an internal control.

### RNA-IP and RNA pulldown assay

RNA-IP was performed as previously described [31]. Briefly, A549 cells (2 × 10^6^) were lysed in Polysome Lysis Buffer (PLB) (100 mM KCl, 5 mM MgCl_2_, 10 mM HEPES (pH 7.0), and 0.5% Nonidet P-40) supplemented with protease inhibitors and 100 U/ml RNAse inhibitor. After centrifugation at 13,000 rpm, the precleared supernatants were incubated with the anti-NP antibody and protein A/G beads for 6 hours at 4°C. After 6 rounds of washing with PLB, the RNA in the bound fractions was eluted using RNAiso plus.

A modified streptavidin-biotin RNA pulldown assay was performed as previously described [31]. Linearized pcDNA WSN NS vRNA was transcribed *in vitro* using T7 polymerase (Promega) and biotin-UTP (Roche). Biotinylated NS vRNA (2 μg) was purified and incubated with cell lysates in a buffer (10 mM Tris-HCl (pH 7.5), 50 mM NaCl, 2 mM MgCl_2_, and 1% Tween-20) supplemented with protease inhibitor and 100 U/ml RNAse inhibitor. Streptavidin agarose resin (Pierce) was added at 4°C for 6 hours. After 6 rounds of washing with incubation buffer, resin-bound proteins were eluted and analyzed by Western blotting.

### Preparation of lipid raft-enriched membrane fractions

Lipid raft-enriched fractions were obtained using a modified TX-100 extraction method [32]. Cells were suspended in prechilled TNE buffer (50 mM Tris-HCl (pH 7.5), 1 mM EDTA, and 150 mM NaCl) with protease inhibitors. After brief sonication, the samples were added to TNE buffer containing 1% TX-100 at 4°C for 30 minutes. The samples were then centrifuged at 13,200 rpm (Eppendorf, 5415R) for 30 minutes to separate the soluble and insoluble fractions.

### Statistics

One-way ANOVA followed by the Newman-Keuls posttest was used to compare differences between three or more independent experimental groups in all cases. A p value <0.05 was considered statistically significant, and individual biological experiments consisted of individual cell culture and virus infection. In general, three or more repeated individual experiments were used for each condition, which was sufficient to determine statistically significant differences.

## Supporting information

S1 Fig Alignment of the WSN NP and NA amino acid sequences compared to those of PR8. (PDF)

S2 Fig Production of recombinant WSN viruses. *In vitro* virus production-related plasmids with indicated the NP and NA plasmids were transfected into 293T cells. Virus production was measured in supernatants by TCA precipitation. The presence of virus or NP-NA proteins in each sample was determined by Western blotting.
(PDF)

S3 Fig Location of NP residues 100 / 373 and NA residues 106 / 248 in the 3D structure. (PDF)

S4 Fig No physical interaction between NP and NA. The indicated NP and NA protein expression plasmids were transfected into 293T cells. Physical interaction between proteins was measured by immunoprecipitation with the anti-NP antibody. Each NP-bound NA protein was determined by Western blotting.
(PDF)

S5 Fig Translocalization to the lipid raft compartment was increased by the NA D248N mutation. The lipid raft-enriched membrane compartment was extracted from 293T cells transfected with the indicated plasmids. The presence of NA protein in each fraction was determined by Western blotting.
(PDF)

S6 Fig NA associated with lipid rafts was reduced by myriocin pretreatment. (A) A549 cells were treated with myriocin (5 μM) or DMSO for 72 hours, and the lipid raft compartment was visualized by fluorescence microscopy after staining with FITC-conjugated cholera-toxin B. (B) A549 cells pretreated with myriocin (5 μM, 48 hours) and then infected with NP^WSN^ NA^WSN^ influenza virus for 24 hours. The level of lipid raft-associated NA protein in the lipid raft fraction was determined by Western blotting.
(PDF)

S1 Table Pearson’s correlation coefficient of amino acid pairs of 2009 pH1N1 IAVs. (PDF)
