# Supplementary material for: Pathogenicity of the H1N1 influenza virus enhanced by functional synergy between the NPV100I and NAD248N pair
Source: PLoS One. 2019 May 31;14(5):e0217691. doi: 10.1371/journal.pone.0217691 (PMC6544299; doi:10.1371/journal.pone.0217691)
Supplement: S1 Fig — (PDF) [file pone.0217691.s001.pdf]

S1 Fig

Virus	NP	NA
PR8	...94 GPIYRRVNGKWMR 106...	...242 MTDGPSDGLASTK 254...
WSN	...94 GPIYRRVDGKWRE 106...	...226 MTDGPSDGLASYK 238...
NP WSN NA WSN	... GPIYRRVDGKWRE ...	... MTDGPSDGLASYK ...
NP V100I NA WSN	... GPIYRRIDGKWRE ...	... MTDGPSDGLASYK ...
NP V100I NA D248N	... GPIYRRIDGKWRE ...	... MTDGPSNGLASYK ...
NP WSN NA D248N	... GPIYRRVDGKWRE ...	... MTDGPSNGLASYK ...
