# Supplementary figures and images for: Pathogenicity of the H1N1 influenza virus enhanced by functional synergy between the NPV100I and NAD248N pair
Source: PLoS One. 2019 May 31;14(5):e0217691. doi: 10.1371/journal.pone.0217691 (PMC6544299; doi:10.1371/journal.pone.0217691)

S2 Fig

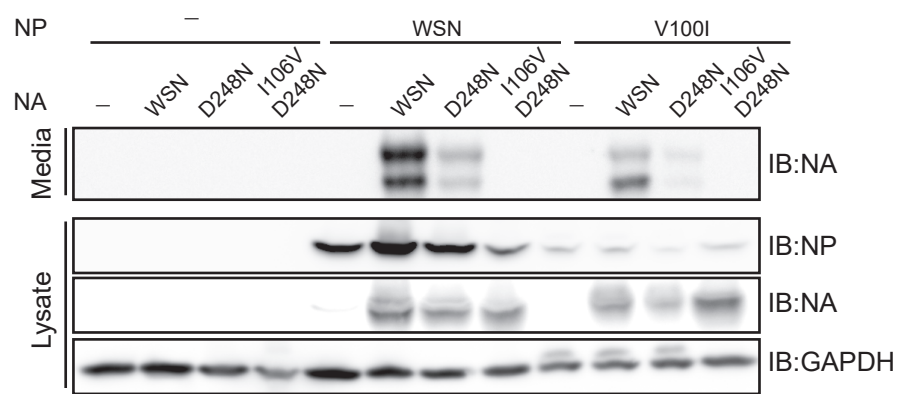

Supplement: S2 Fig — In vitro virus production-related plasmids with indicated the NP and NA plasmids were transfected into 293T cells. Virus production was measured in supernatants by TCA precipitation. The presence of virus or NP-NA proteins in each sample was determined by Western blotting. (PDF) [file pone.0217691.s002.pdf]

S3 Fig

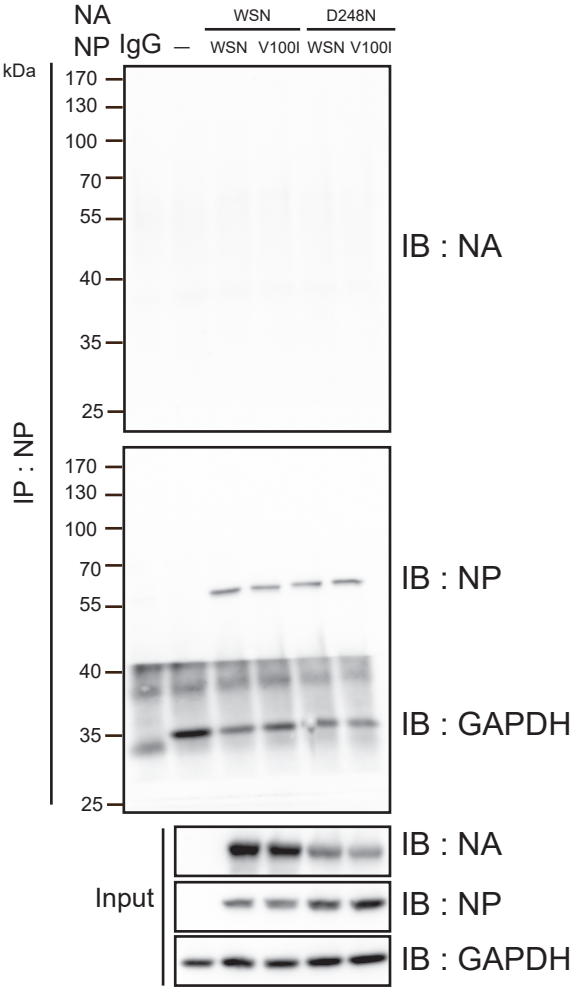

Supplement: S3 Fig — (PDF) [file pone.0217691.s003.pdf]

# S4 Fig

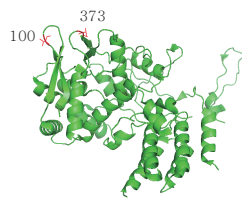

NP

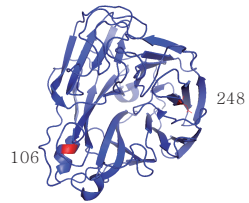

NA

Supplement: S4 Fig — The indicated NP and NA protein expression plasmids were transfected into 293T cells. Physical interaction between proteins was measured by immunoprecipitation with the anti-NP antibody. Each NP-bound NA protein was determined by Western blotting. (PDF) [file pone.0217691.s004.pdf]

# S5 Fig

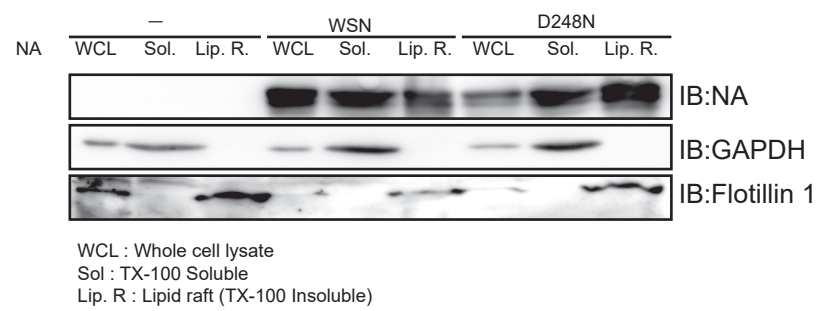

Supplement: S5 Fig — The lipid raft-enriched membrane compartment was extracted from 293T cells transfected with the indicated plasmids. The presence of NA protein in each fraction was determined by Western blotting. (PDF) [file pone.0217691.s005.pdf]

S6 Fig

A

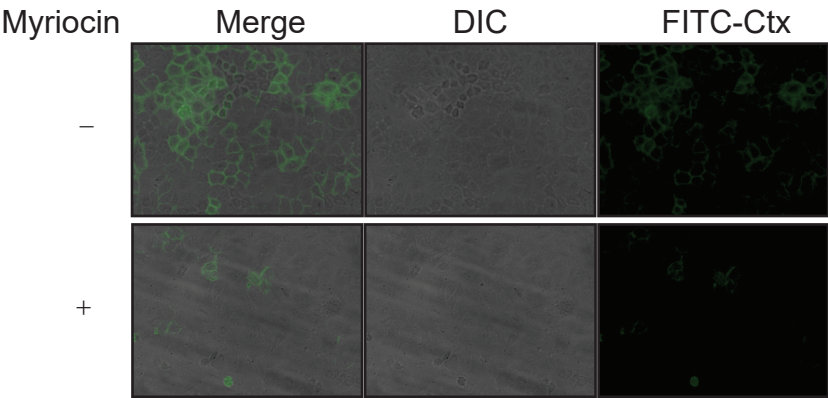

B

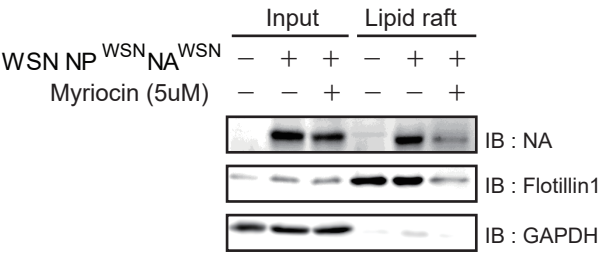

Supplement: S6 Fig — (A) A549 cells were treated with myriocin (5 μM) or DMSO for 72 hours, and the lipid raft compartment was visualized by fluorescence microscopy after staining with FITC-conjugated cholera-toxin B. (B) A549 cells pretreated with myriocin (5 μM, 48 hours) and then infected with NPWSN NAWSN influenza virus for 24 hours. The level of lipid raft-associated NA protein in the lipid raft fraction was determined by Western blotting. (PDF) [file pone.0217691.s006.pdf]
