# Supplementary material for: Pathogenicity of the H1N1 influenza virus enhanced by functional synergy between the NPV100I and NAD248N pair
Source: PLoS One. 2019 May 31;14(5):e0217691. doi: 10.1371/journal.pone.0217691 (PMC6544299; doi:10.1371/journal.pone.0217691)
Supplement: S1 Table — (PDF) [file pone.0217691.s007.pdf]

S1 Table

Amino acid		
Gene X	Gene Y	Pearson's *r*
NP	NA	0.623
PB1	NA	0.253
PB2	PB1	0.246
HA	NA	0.239
HA	NS1	0.205
PB1	NP	0.201
PB2	NA	0.201
HA	NP	0.189
PB1	NS2	0.153
PA	NS1	0.136
PB2	NP	0.132
PA	M2	0.131
HA	M1	0.125
PB2	NS2	0.118
NA	NS1	0.104
M1	NS2	0.103
HA	M2	0.090
PB2	PA	0.087
PB2	HA	0.086
PB1	HA	0.081
PA	NP	0.080
NS1	NS2	0.075
NA	NS2	0.072
NP	M2	0.055
NP	NS1	0.048
NA	M2	0.039
HA	NS2	0.037
PB2	NS1	0.034
PA	NA	0.033
PB1	M2	0.029
PA	M1	0.026
PB2	M1	0.022
M1	M2	0.015
PA	HA	0.014
PB1	M1	0.012
PB1	PA	0.012
NP	NS2	0.010
PA	NS2	-0.016
NP	M1	-0.016
M2	NS1	-0.020
M1	NS1	-0.025
M2	NS2	-0.025
PB2	M2	-0.026
NA	M1	-0.035
PB1	NS1	-0.104
